# Nitrogen Fertilization Restructured Spatial Patterns of Soil Organic Carbon and Total Nitrogen in Switchgrass and Gamagrass Croplands in Tennessee USA

**DOI:** 10.1038/s41598-020-58217-x

**Published:** 2020-01-27

**Authors:** Jianwei Li, Siyang Jian, Chad S. Lane, Chunlan Guo, YueHan Lu, Qi Deng, Melanie A. Mayes, Kudjo E. Dzantor, Dafeng Hui

**Affiliations:** 1 Department of Agricultural and Environmental Sciences, Tennessee State University, Nashville, TN 37209 USA; 2 Department of Earth and Ocean Sciences, University of North Carolina Wilmington, Wilmington, NC 28403 USA; 3 Jiangxi Provincial Key Laboratory for Bamboo Germplasm Resources and Utilization, Forestry College, Jiangxi Agricultural University, Nanchang, 330045 Jiangxi China; 4 Department of Geological Sciences, University of Alabama, Tuscaloosa, Alabama 35487 USA; 5 Key laboratory of Vegetation Restoration and Management, South China Botanical Garden, The Chinese Academy of Sciences, Guangzhou, 510650 China; 6 Climate Change Science Institute and Environmental Sciences Division, Oak Ridge National Laboratory, Oak Ridge, Tennessee 37831 USA; 7 Department of Biological Sciences, Tennessee State University, Nashville, TN 37209 USA

**Keywords:** Element cycles, Carbon cycle

## Abstract

Nitrogen (N) fertilizers can potentially alter spatial distribution of soil organic carbon (SOC) and total nitrogen (TN)  concentrations in croplands such as switchgrass (SG: *Panicum virgatum* L.) and gamagrass (GG: *Tripsacum dactyloides* L.), but it remains unclear whether these effects are the same between crops and under different rates of fertilization. ^13^C and ^15^N are  two important proxy measures of soil biogeochemistry, but they were rarely examined as to their spatial distributions in soil. Based on a three-year long fertilization experiment in Middle Tennessee, USA, the top mineral horizon soils (0–15 cm) were collected using a spatially explicit design within two 15-m^2^ plots under three fertilization treatments in SG and GG croplands. A total of 288 samples were collected based on 12 plots and 24 samples in each plot. The fertilization treatments were no N input (NN), low N input (LN: 84 kg N ha^−1^ in urea) and high N input (HN: 168 kg N ha^−1^ in urea). The SOC, TN, SOC/TN (C: N), δ^13^C and δ^15^N were quantified and their within-plot variations and spatial distributions were achieved via descriptive and geostatistical methods. Results showed that SG generally displayed 10~120% higher plot-level variations in all variables than GG, and the plot-level variations were 20~77% higher in NN plots than LN and HN plots in SG but they were comparable in unfertilized and fertilized plots in GG. Relative to NN, LN and HN showed more significant surface trends and spatial structures in SOC and TN in both croplands, and the fertilization effect appeared more pronounced in SG. Spatial patterns in C: N, δ^13^C and δ^15^N were comparable among different fertilization treatments in both croplands. The descending within-plot variations were also identified among variables (SOC > TN > δ^15^N > C: N > δ^13^C). This study demonstrated that N fertilizations generally reduced the plot-level variance and simultaneously re-established spatial structures of SOC and TN in bioenergy croplands, which little varied with fertilization rate but was more responsive in switchgrass cropland.

## Introduction

As two important bioenergy crops, perennial switchgrass (SG: *Panicum virgatum* L) and gamagrass (GG: *Tripsacum dactyloides* L) are common alternative energy sources for sustainable replacement of fossil fuels^1–5^. N fertilizers are widely amended to increase bioenergy crop yields^6–8^, but N fertilization impact on soil organic C (SOC) and total soil N (TN) contents varied in magnitude and signal^9–11^. The large variability of fertilization effects among different studies are seldom attributed to the underlying variations in space. In fact, the generally very low number of soil samples in a study (i.e., 3~5) inevitably amplified the influence of spatial variations on the detected treatment effect^12^. Furthermore, the crop-specific root morphology and chemistry largely regulated the spatial variation of soil biogeochemistry^13,14^ and may have contributed to contrasting SOC and TN stocks and their responses to N fertilization between SG and GG^15^. Thus, elucidating the effects of fertilization on spatial distribution of soil C and N will provide fundamental knowledge needed to develop effective strategies to improve soil quality, C sequestration, agricultural productivity, and climate change adaptation^16–19^.

A limited number of studies however have focused on the spatial distributions of soil C and N in bioenergy croplands. In a SG cropland, the spatial dependence of SOC and TN at soil depth of 0–30 cm is weak to moderate when the point-to-point distance is less than 200 m^20^. This contrasts with the observation that a strong spatial dependence of SOC and TN exists within 30 m in a loblolly pine ecosystem^21^. As the major driver of soil organic matter decomposition, soil microbial communities responded strongly to 3-year N fertilization such that N fertilizer inputs enhanced the spatial heterogeneity of microbial biomass C and N in bioenergy croplands^22^. It is expected that the generated hotspots of microbial communities in a fertilized plot may result in greater soil C and N accumulations due to fast microbial necromass incorporated into soil organic matter pool^23–25^. Besides the elevated microbial communities, higher earthworm activity, soil porosity, water holding capacity, and soil aeration as well as organic carbon storage were also revealed with decade to century long fertilizations^26^. Because N fertilization impacts on crop productivity and microbial activities occurred annually or over even a shorter time scale, this may likely shift spatial distribution and structure of soil C and N over month to year time scales. Such evidence is however rarely reported. Although many studies reported the effects of agricultural practices on the stocks and spatial patterns of soil C and N in forests and cultivated lands^27–29^, such studies are surprisingly scarce in bioenergy croplands. In these studies, effect of N fertilization was always masked due to its interaction with other agricultural practices (e.g., tillage and irrigation). It remains largely unknown whether the fertilization effects will depend upon the duration of fertilization.

Soil δ^13^C and δ^15^N are widely used to infer vegetation succession, SOC dynamics, and nutrient leaching in bioenergy croplands^30–32^, the spatial variability of these biogeochemical tracers has yet to be studied in SG or GG. In a subtropical woodland, the distance of dissimilar δ^13^C in soil appears beyond 15.6 m (i.e., spatial autocorrelation), which is far greater than individual tree canopy diameter in these lowland communities^33^. TN and δ^15^N are likely to display more spatial variations due to relative low concentrations and diverse chemical forms^34^, but these have not been confirmed yet. We speculate that the N fertilizer induced greater input to soil via litterfall and root exudate may have contributed to altered spatial variations of microbial biomass as observed formerly^22^, and this can further stimulate microbial mineralization of more ^13^C enriched and older soil organic matter^35,36^. This speculation will not only decrease δ^13^C values in soil under N fertilization, which resulted from greater release of ^13^C via heterotrophic respiration of old-aged soil^37^, but also change the spatial distribution of ^13^C. However, no studies have reported the spatial variations of soil δ^13^C and δ^15^N in different bioenergy croplands and under different N fertilization treatments.

Using a bioenergy crop field experiment in Middle Tennessee, we investigate the effects of N fertilization on the plot-level variances and spatial patterns in elemental and stable isotopic characteristics of soil C and N in two bioenergy croplands (SG and GG). N fertilization input represents the primary management practice in our research plots and no tillage, plowing, or minor mechanical disturbance was applied. We hypothesize that relative to those soils that have not been fertilized for 3 years, continued N fertilization at either low or high rates (84 and 168 kg N ha^−1^) will reduce the within-plot variances but restructure the spatial patterns of SOC, TN, C:N, δ^13^C, and δ^15^N; Furthermore, these aforementioned fertilization effects little depend upon fertilization rate or crop type.

## Material and Methods

### Site description and characteristics

**Figure 1:**
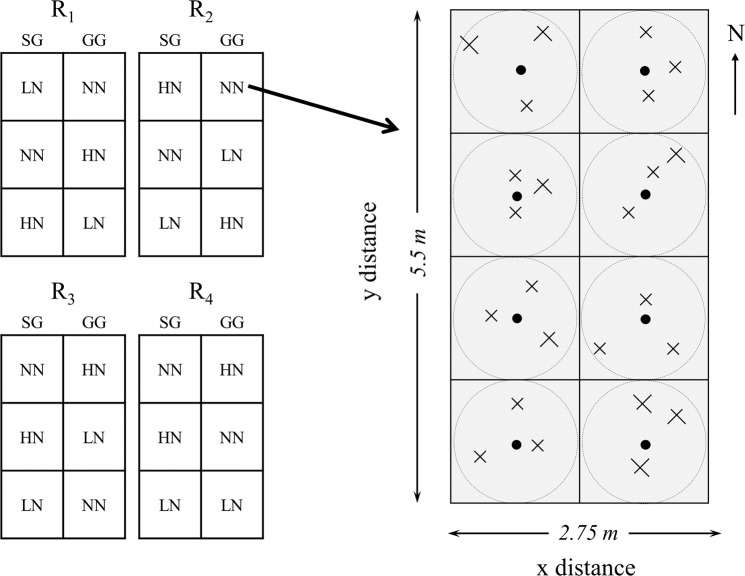
Left: the diagram of the experiment plots in the three-year long fertilization experimental site at the Tennessee State University (TSU) Agricultural Research Center in Nashville, TN, USA. Two of four blocks (R1, R2) were sampled in this study. Right: illustration of a clustered random sampling design in a plot. Filled circles represent centroids (n = 8) and each plot consists of eight centroids with one in subplot (1.375 m × 1.375 m). In each subplot, a circular was determined for soil sampling. Xs represent sample locations determined from random directions and distances from a centroid within each circular sampling area (dashed circle). The extent of an interpolation map was thus determined by the minimum and maximum values at horizontal and vertical axes, and each map can attain its extent less than or equivalent to the study area (2.75 m × 5.5 m rectangle).

Prior to the switchgrass and gamagrass croplands, the land use is the mowed grassland for decades. No fertilizers have been applied in the prior land use. Therefore, the indigenous variations are similar in both croplands. Initially established in 2011, the bioenergy crop field experiment is located at the Tennessee State University (TSU) Main Campus Agriculture Research and Education Center (AREC) in Nashville, TN, USA (Lat. 36.12° N, Long. 36.98° W, elevation 127.6 m above sea level). Climate in the region is a warm humid temperate climate with an average annual temperature of 15.1 °C, and total annual precipitation of 1200 mm^38^. Two crop types and three N fertilization levels were included in a randomized block design^22,39^. The two crop types were *Alamo* SG (*Panicum virgatum* L.) and GG (*Tripsacum dactyloides* L.). The three N levels included no N fertilizer input (NN), low N fertilizer input (LN: 84 kg N ha^−1^ yr^−1^ as urea), and high N fertilizer input (HN: 168 kg N ha^−1^ yr^−1^ as urea), and each treatment had four replicated plots with a dimension of 3 m × 6 m (Fig. 1). The low N fertilization rate represented the agronomically optimum nitrogen rate to maximize cellulosic ethanol production in established northern latitude grasslands^40^. The high N rate doubled the low rate in order to create appreciable gap and detectable effect between the two levels. The fertilizer was manually applied in June or July each year after cutting the grass. Fertilizer was applied after cutting grass during June to July each year. The soil series for the plots is Armour silt loam soil (fine-silty, mixed, thermic Ultic *Hapludalfs*) with acidic soil pH (i.e., 5.97) and intermediate organic matter content of 2.4%^22,41^.

### Soil collection and laboratory analysis

We collected soil samples (0–15 cm) from 12 plots (2 crops × 3 N inputs × 2 replicated plots) on June 6, 2015. Within each plot, we divided a 2.75– m × 5.5– m rectangular zone into eight equal-sized square subplots. Within each subplot, a centroid was established and a circular sampling zone was created with the diameter equivalent to side length of the square (Fig. 1). Three cores were randomly collected within each circular sampling zone. Relative to the origin at the southwestern corner in each plot, the distances at the horizontal and vertical directions of each soil core were measured and assigned to each soil core as *x*, *y* coordinates. A total of 288 soil cores were obtained based on 24 cores in each of 12 plots. Similar clustered soil sampling approaches were described in formerly published work^12,21,22^. The soil samples were transported to the TSU lab in a cooler filled with ice packs and were then subsequently stored at 4 °C until analysis. Visible roots and rocks were removed from the samples, and soil samples were then passed through a 2 mm soil sieve. The air-dried soil subsamples were ground and homogenized to fine powder for the analysis of SOC, TN, stable carbon, and nitrogen isotopic compositions (δ^13^C, δ^15^N). The analysis was conducted at the University of North Carolina Wilmington Isotope Ratio Mass Spectrometry facility using a Costech 4010 elemental analyzer interfaced with a Thermo Delta V Plus stable isotope mass spectrometer.

### Descriptive and geospatial analysis

The frequency distribution, within-plot variance, plot level coefficient of variation (CV), and sample size requirement were calculated to describe plot level variances^21,22^. The Pearson Moment correlation coefficients were derived among SOC, TN, C: N, microbial biomass carbon (MBC), microbial biomass N (MBN) and MBC: MBN (C: N_mb_). The microbial biomass data were adapted from the previous publication^22^. The study derived the sample size requirement (*N*) in each plot given specified relative errors (γ, 0~100%) in order to evaluate how within-plot variances (i.e., sample size requirements) are altered by N fertilization or crop type at certain relative error.

1 $$CI=\bar{X}\pm {t}_{0.975}\times \frac{s}{\sqrt{n}}$$

2 $$\gamma =\frac{{t}_{0.975}\times \frac{s}{\sqrt{N}}\,}{\bar{X}}={t}_{0.975}\times \frac{CV}{\sqrt{N}}$$

3 $$\mathrm{ln}({\rm{N}})=2\times \,\mathrm{ln}({t}_{0.975}\times {\rm{CV}})-2\times \,\mathrm{ln}({\rm{\gamma }})$$

Where CI, $\overline{X,}$ *s*, *n*, *N*, *CV*, and γ denote confidence interval, plot mean, plot standard deviation, sample number (n = 24), coefficient of variation, sample size requirement and relative error, respectively. *t*_0.975_ = 1.96. The log transformed sample size requirement (*N*) has a negative linear relationship (i.e. slope = 2) with the log transformed relative error (γ).

Cochran’s C test is used to test the assumption of variance homogeneity. The test statistic is a ratio that relates the largest empirical variance of a particular treatment to the sum of the variances of the remaining treatments. The theoretical distribution with the corresponding critical values can be specified^42–44^. Soil properties that exhibited non-normal distributions were log-transformed to better conform to the normality assumption of the Cochran’s C test^21^. The variances were compared between all plots under three fertilization treatments for each crop and among two crops. When p-value is < 0.05 for the comparison conducted in each crop, the largest variance was identified.

Three different geostatistical approaches were used to display the spatial pattern, structure and distribution of all variables within and among plots and they include trend surface analysis (TSA), Moran’s I index for spatial autocorrelation, and inverse distance weighting (IDW) for distribution map. Details can be found in former publications^21,22^. Brief introduction of the three geostatistical approaches were also attached. To note, the plot-level variation (i.e., CV) was calculated based on the 24 measurements of soil samples randomly collected in a plot. By accounting for their respective spatial coordinates and distance among samples, the same number of measurements was also used to derive the spatial variability (i.e., spatial heterogeneity) via the three different geostatistical methods. Therefore, there is no direct correlation between the spatial variability (i.e., spatial heterogeneity) and plot-level variation (i.e., CV).

First, the trend surface analysis (TSA) is the most common regionalized model in which all sample points fit a model that accounts for the linear and non-linear variation of an attribute. The relationships between the soil properties and the x and y coordinates of their measurement location within the sampling plots are estimated with the trend surface model:

4 $${\rm{Soil}}\,{\rm{property}}\,{\rm{value}}={\beta }_{0}+{\beta }_{1}x+{\beta }_{2}y+{\beta }_{3}xy+{\beta }_{4}{x}^{2+}{\beta }_{5}{y}^{2}$$

with regression coefficients β_0_ to β_5_. The presence of a trend in the data was determined by the significance of any of the parameters β_1_ to β_5_, while the β_0_ term modeled the intercept^45,46^. Linear gradients in the x or y directions were indicated by significance of the β_1_ or β_2_ parameters. A significant β_3_ term indicated a significant diagonal trend across a plot. Significant β_4_ and β_5_ parameters indicated more complex, nonlinear spatial structure such as substantial humps or depressions. Trend surface regressions were estimated using S-plus (version 7.0, Insightful Inc.). Model parameters were determined to be significant at a level of *P* < 0.05. The number of significant parameters was counted for each plot and compared among treatments.

Second, residuals from the trend surface regressions were saved for subsequent spatial analysis using a Moran’s I index^46^. The Moran’s I analysis^47–49^ was used to quantify the degree of spatial autocorrelation that existed among all soil cores taken from each plot. The resulting local Moran’s I statistics are in the range from –1 to 1. Positive Moran’s I values indicate similar values (either high or low) are spatially clustered. Negative Moran’s I values indicate neighboring values are dissimilar. Moran’s I values of 0 indicate no spatial autocorrelation, or spatial randomness. A significant autocorrelation is determined if the observed Moran’s I value is beyond the projected 95% confidence interval at certain distance. Correlograms for local Moran indices were estimated for each soil variable in each plot in a range of 0–5.5 meter with 0.5 meter incremental interval. The number of distance corresponding to the significant Moran’s I was counted for each plot and compared among treatments.

Third, due to relatively small sample sizes (n = 24) per plot^50^, we used inverse distance weighting (IDW) interpolation rather than ordinary kriging^51^. The maps produced by IDW offered direct and visual assessments from which to compare the spatial distributions of the soil properties among the plots. The IDW method derived maps was able to distinguish effects of different land uses on spatial distributions of soil biogeochemical features in South Carolina, USA^21^. The weights for each observation are inversely proportional to a power of its distance from the location being estimated. Exponents between 1 and 3 are typically used for IDW, with 2 being the most common^52^. Tests with different IDW exponents indicated that 2 was optimal with these data, as estimated values generated with an exponent of 2.0 showed the best fit with actual data in cross validation tests. ArcGIS 9.0 was used to generate the IDW maps and perform cross validations.

## Results

### Frequency distribution, plot-level variance, CV and sample size requirement

The frequency distributions of all variables appeared concentrated in relatively narrow ranges across different fertilization treatments (Fig. S1). The concentrated ranges of SOC and TN were shifted to greater values under HN and LN relative to NN, but substantial overlaps between fertilization treatments were identified for other variables (Fig. S1). The within-plot variances generally appeared the highest in one of NN plots for all variables in SG, and in one of LN or HN plots for SOC and C: N in GG (Table 1). Only exception is δ^13^C in SG with the highest within-plot variance in one of HN plots meanwhile other plots of HN and LN were at least two times lower than that for NN plots (Table 1). The within-plot variances appeared insignificantly different between fertilization treatments for TN, δ^13^C and δ^15^N (Table 1).

**Table 1:** Plot-level variance and Cochran’s C test results for SOC and TN, C: N, δ^13^C, and δ^15^N from three N fertilization treatments (i.e., NN, LN and HN) in SG and GG.

Crop	Fertilization	Plot	SOC	TN	C:N	δ^13^C	δ^15^N
SG	NN	P1	0.05	0.0002	**1.59**	1.25	0.25
		P2	**0.08**	**0.0005**	0.23	1.32	**0.52**
	LN	P1	0.03	0.0002	0.39	0.61	0.16
		P2	0.05	0.0004	0.12	0.58	0.24
	HN	P1	0.06	0.0003	0.30	**3.34**	0.10
		P2	0.04	0.0002	0.14	0.41	0.09
Cochran’s *C* test		*C* value	0.25	0.27	0.57	0.45	0.39
		*p-*value	*0.04*	*0.01*	*0.00*	*0.00*	*0.00*
GG	NN	P1	0.02	0.0002	0.17	0.24	0.09
		P2	0.06	0.0004	0.34	0.36	0.11
	LN	P1	0.03	0.0003	**0.53**	0.39	0.12
		P2	0.05	0.0002	0.19	0.67	0.10
	HN	P1	**0.09**	0.0003	0.37	0.79	0.08
		P2	0.04	0.0002	0.22	0.40	0.12
Cochran’s C test		*C* value	0.31	0.23	0.29	0.28	0.19
		*p-*value	*0.02*	0.44	*0.04*	0.08	1.00
Total Cochran’s *C* test		*C* value	0.14	0.14	0.35	0.32	0.26
		*p-*value	0.13	0.18	*0.00*	*0.00*	*0.00*

Abbreviations: SG (switchgrass), GG (gamagrass), NN (no fertilizer input), LN (low fertilizer input, 84 kg N ha^−1^), and HN (high fertilizer input, 168 kg N ha^−1^).

The italic numbers denote *p-value* < 0.05.

The bond numbers denote the largest among all plots for each crop when *p*-value is less than 0.05.

**Figure 2:**
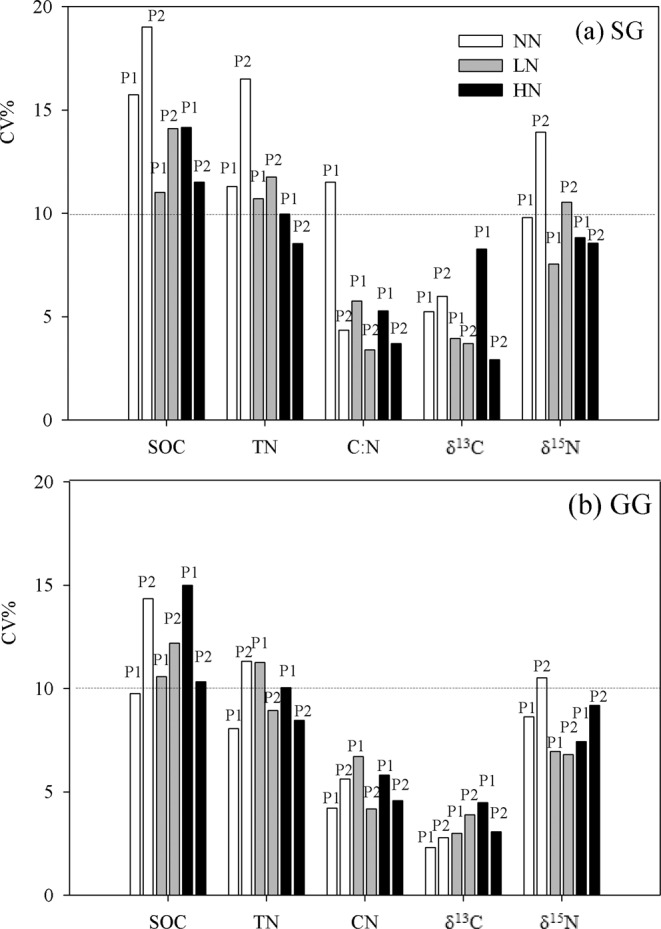
Within-plot CVs of SOC and TN, C: N, δ^13^C, and δ^15^N under three N fertilization (i.e. NN, LN and HN) in (**a**) SG and (**b**) GG in a three-year long fertilization experimental site at the Tennessee State University (TSU) Agricultural Research Center in Nashville, TN, USA. The line represents CV of 10% in both panels.

The within-plot CVs were less than 20% for all variables in both croplands and were generally larger in SG than that in GG (Fig. 2). The within-plot CVs were less than 10% for all plots of δ^13^C in both croplands, C: N in GG, and most plots C: N in SG and δ^15^N in both croplands. The within-plot CVs varied among different variables and followed a descending order as SOC > TN > δ^15^N > C:N > δ^13^C (Fig. 2). The within-plot CV differed less pronouncedly among fertilization treatments for GG (Fig. 2). The highest CV was 19.0% for SOC in SG and the lowest 2.3% for δ^13^C in GG. The average CV of two plots under NN was 20~77% higher than that under LN or HN in SG (Fig. 2a), not in GG (Fig. 2b). The within-plot CV appeared the largest in one of NN plots for SG (except δ^13^C).

**Figure 3:**
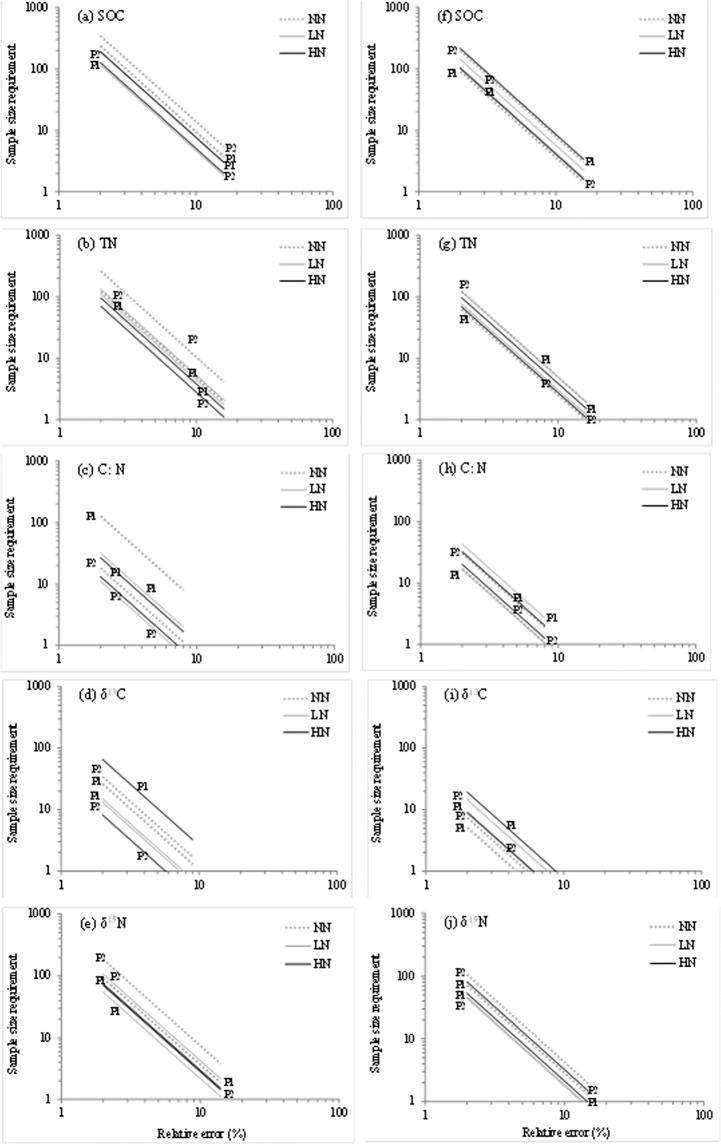
The plotted linear regression lines between log transformed sample size requirements (SSR) and desired relative errors (%) for SOC and TN, C: N, δ^13^C and δ^15^N in SG (left column) and GG (right column) in a three-year long fertilization experimental site at the Tennessee State University (TSU) Agricultural Research Center in Nashville, TN, USA. The log scale was applied on both axes.

Between crops, the plotted lines of sample size requirement against relative sampling error departed from one another more pronouncedly among plots in SG than in GG (comparing the left and right panels, Fig. 3). Among different variables, a larger number of samples were required for SOC and TN than for C: N, δ^13^C, and δ^15^N given the same desired relative error (Fig. 3). Among fertilization treatments, the largest number of samples were required for one of NN plot in SG (except δ^13^C), and only for TN and δ^15^N in GG.

Although the mean changes of these variables were not the focus in the current study, the summary statistics (e.g., min, max, range, mean, SE, SD etc.,) were presented in Table S1. The current study focused on the spatial patterns and thus the summary results will be discussed in another relevant publication under preparation.

### Trend surface analysis

More surface trends of SOC, TN and C:N, shown either in the x or y directions, diagonal direction, or as nonlinear spatial structure such as substantial humps or depressions, were significant in the fertilized plots (i.e., LN, HN or both) than unfertilized plots in both crops (Table 2). Surface trends of δ^13^C were not significant in all plots, and the number of significant surface trends of δ^15^N appeared comparable in all treatments in SG; However, the number of surface trends of δ^13^C and δ^15^N were comparable in NN and LN but not in HN in GG (Table 2). No difference was identified between the two crops.

**Table 2:** Significant regression coefficients for trend-surface analysis and coefficients of determination (*r*^2^) in SOC and TN, C:N, δ^13^C, and δ^15^N from three N fertilization treatments (i.e., NN, LN, and HN) in SG and GG. The regression coefficients are used to describe whether a significant decreasing or increasing trend exist. Details are in Li *et al*. (2010). *, ** and ***represent significance at P < 0.05, 0.01 and 0.001, respectively.

Crop	Variable	Fertilization	Plot	β0	β1x	β2y	β3x²	β4y²	β5xy	r²
SG	SOC	NN	P1	1.55***						0.21
			P2	1.19**						0.25
		LN	P1	1.33***						0.30
			P2	1.39***				−0.05*		0.38
		HN	P1	1.15**		0.31*			−0.10*	0.44
			P2	1.83***						0.19
	TN	NN	P1	0.17***					0.006*	0.39
			P2	0.11**						0.23
		LN	P1	0.13***						0.33
			P2	0.14***						0.39
		HN	P1	0.12***		0.02**			−0.006*	0.50
			P2	0.17***						0.17
	C:N	NN	P1	8.96***						0.14
			P2	11.0***						0.39
		LN	P1	10.0***						0.40
			P2	10.03***						0.23
		HN	P1	9.38***					−0.23*	0.31
			P2	10.82***						0.22
	δ^13^C	NN	P1	−21.90***						0.15
			P2	−19.90***						0.11
		LN	P1	−20.02***						0.22
			P2	−19.44***						0.52
		HN	P1	−24.17***						0.29
			P2	−21.41***						0.24
	δ^15^N	NN	P1	4.02***		0.89**		−0.12**		0.52
			P2	6.06***				−0.17**		0.65
		LN	P1	4.22***		0.58*		−0.08*		0.48
			P2	4.83***					−0.13*	0.68
		HN	P1	4.43***		−0.39**			0.10**	0.75
			P2	3.06***						0.11
GG	SOC	NN	P1	1.62***						0.28
			P2	1.58***						0.34
		LN	P1	1.87***						0.19
			P2	2.11***						0.46
		HN	P1	2.07***						0.35
			P2	1.19***	0.65*					0.39
	TN	NN	P1	0.17***						0.28
			P2	0.18***						0.41
		LN	P1	0.17***		−0.03*		0.003*		0.30
			P2	0.20***						0.49
		HN	P1	0.19***						0.41
			P2	0.14***						0.47
	C:N	NN	P1	9.70***				0.11*		0.42
			P2	8.74***						0.26
		LN	P1	10.78***		1.10*		−0.20**		0.40
			P2	10.71***						0.30
		HN	P1	10.82***						0.26
			P2	8.97***	2.02*		−0.60*			0.37
	δ^13^C	NN	P1	−22.32***						0.37
			P2	−23.33***						0.50
		LN	P1	−20.14***	−1.86**			−0.14**	0.28**	0.72
			P2	−19.35***		−1.34***		0.14*		0.69
		HN	P1	−20.64***						0.38
			P2	−22.03***						0.20
	δ^15^N	NN	P1	3.29***						0.09
			P2	2.91***		0.45*		−0.07*		0.44
		LN	P1	3.58***	1.29*	0.50*	−0.35*			0.38
			P2	5.10***						0.25
		HN	P1	3.65***						0.17
			P2	4.58***						0.49

### Spatial autocorrelation

Spatial autocorrelations for SOC and TN were not identified at any distance in NN plots, but did show at different distances in LN and HN plots in SG (Table 3; Fig. S2). Spatial autocorrelations for SOC and TN were more frequently identified in either LN or HN than NN in GG. The comparable spatial autocorrelations for C: N, δ^13^C, and δ^15^N were identified in both SG and GG (Table 3; Fig. S2). These significant autocorrelations were either positive or negative, and the lagging distances ranged from 0.50 m to 4.75 m. Spatial autocorrelations for SOC, TN, C: N, and δ^13^C were generally more frequently identified in GG than SG but the opposite was true for spatial autocorrelations for δ^15^N (Table 3).

**Table 3:** Summary of significant distance for spatial dependence based on Moran^’^s *I* analysis for SOC and TN, C:N, δ^13^C, and δ^15^N from three N fertilization treatments (i.e., NN, LN, and HN) in SG and GG.

Crop	Fertilization	Plot	SOC	TN	C:N	δ^13^C	δ^15^N
					meter		
SG	NN	P1			0.5		0.5, −2.25, −4.5, −4.75
		P2			−4.75	3.75, 4.5	0.75, 1
	LN	P1	3.5		−1.25	−4.25	1, 1.5, −2.75, −3, −3.25
		P2	−2.75, −3.25	−2.75	3.75	−3.75	0.75, −3.75, −4.25, −4.5
	HN	P1	0.5, −2	0.5, −2			0.75, 1, 1.5, −3.75, −4, −4.75, −5
		P2	−1.75	−2.75	−1.75	−4	−1
GG	NN	P1	1.25	1.25	−3, 4.25		2.25
		P2	−3.75, 4.25, −4.5	−3.75, −4.5, −5	−2	0.75, −4.25, −4.5	−3.5, −3.75, −4.5, −4.75
	LN	P1		−3.5	4.75	0.75, −3, −3.75	0.5, −1.75
		P2	−0.75, −3.75, −4.25, −4.75	0.75, 1.5, −3.75, −4.25, −4.75	0.75, −3.75, −4.25	0.75, 1.25, −3.75, −4, −4.25	−2.75
	HN	P1	1.25, 1.75		1.75, 2.5	2.5	1.75, −3.75
		P2	−4.25			−1.5	0.75, 4.25, 4.75

### Distribution map via inverse distance weighting method

**Figure 4:**
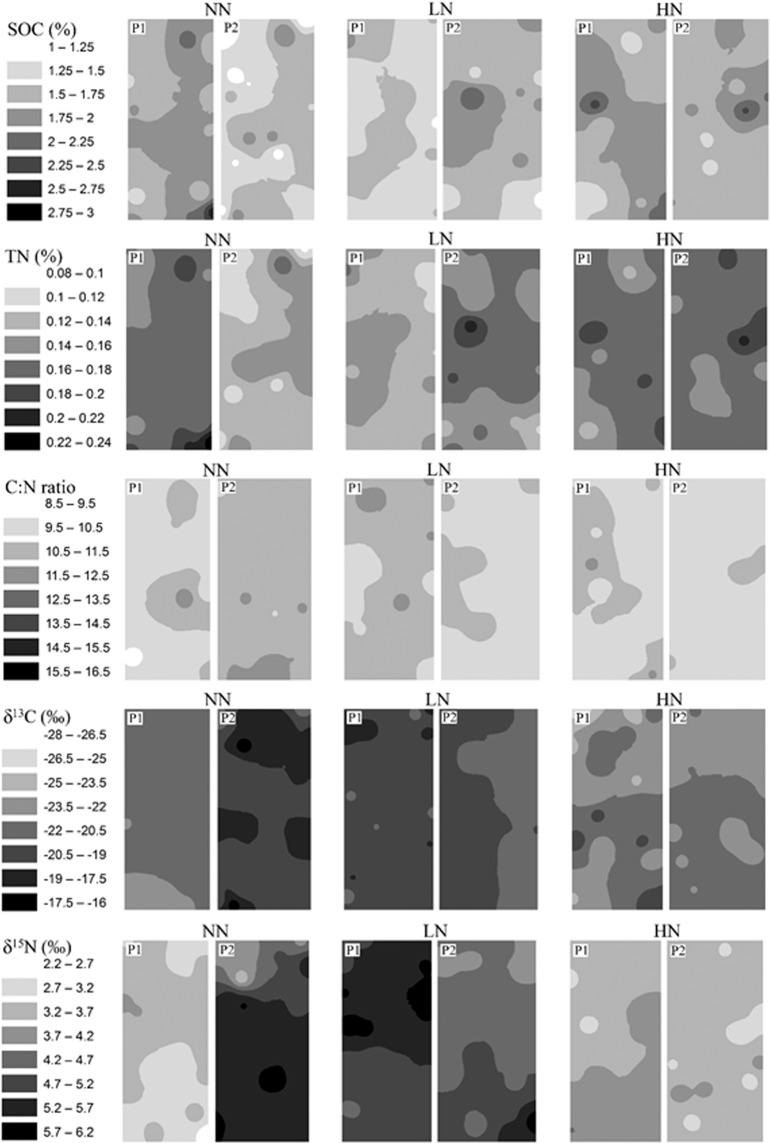
Inverse distance weighted (IDW) maps of SOC, TN, C: N, δ^13^C, and δ^15^N in two plots (P1, P2) under three N fertilization treatments (i.e. NN, LN and HN) in SG cropland soils in a three-year long fertilization experimental site at the Tennessee State University (TSU) Agricultural Research Center in Nashville, TN, USA.

**Figure 5:**
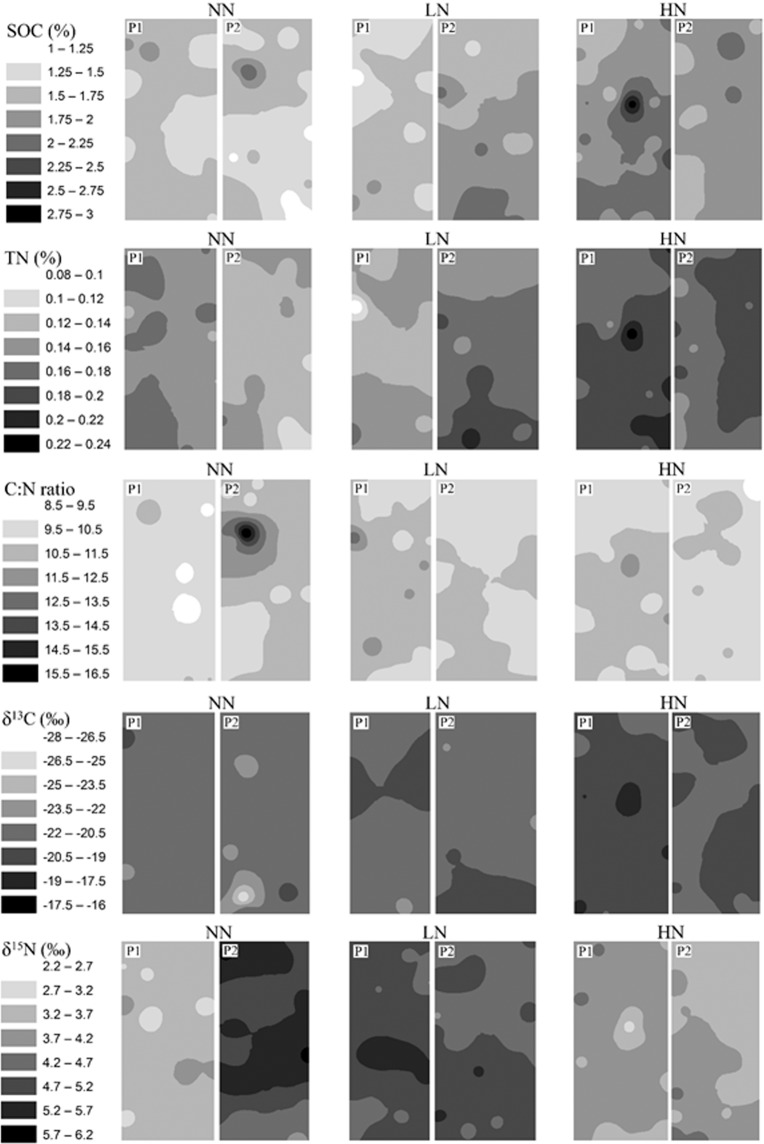
Inverse distance weighted (IDW) maps of SOC, TN, C: N, δ^13^C, and δ^15^N in two plots (P1, P2) under three N fertilization treatments (i.e. NN, LN and HN) in GG cropland soils in a three-year long fertilization experimental site at the Tennessee State University (TSU) Agricultural Research Center in Nashville, TN, USA.

Based on the spatial distribution maps of SOC and TN, those in fertilized plots (particularly HN) exhibited darker color (i.e., higher concentration) and more evidence of spatial structures than NN plots in both croplands (Figs. 4 and 5). In contrast, C: N, δ^13^C, and δ^15^N exhibited substantial plot-to-plot variations under the same treatment, particularly for the two plots under NN in both croplands (Figs. 4 and 5). The plot-to-plot differences of the distribution maps were reduced under LN and HN in both croplands (Figs. 4 and 5).

## Discussion

### N fertilization reduced the plot-level variations of soil variables in SG

Our results demonstrated that both low and high fertilization rates generally reduced the average variances and CVs across two plots in all variables in SG. However, these variations were little changed with N fertilization in GG. This was likely associated with the much higher variations in SG than in GG (i.e., under NN; Fig. 2), reflecting differential impact of plant species in regulating plot-level variations when N fertilizer was absent. Furthermore, this revealed that the substantial reduction in variance, i.e., greater responsiveness of SG than GG may be driven by their contrasting plant root chemistry and morphology such that the more labile and higher bioreactivity of SG root (unpublished data) is more sensitive to N fertilizer resulting in much faster root turnover and potentially homogenization of originally more varied soil features. Interestingly, the plot-level CVs of the same variable appeared similar under N fertilized plots in both croplands, e.g., 10~15% of CV for SOC, which likely represented a unique and common characteristics of soil biogeochemistry for the bioenergy crops due to their massive root system and high crop yield^53,54^. Meanwhile, it was also apparent that large plot to plot variations under the same treatment existed, e.g., CVs of most variables under NN in SG. In fact, relative to fertilized plots, the enormously larger plot-level variance always appeared in only one but not in another unfertilized plot. This suggested that despite a generally high variation of soil biogeochemistry in unfertilized bioenergy croplands, other factors such as sampling time and location, crop species and variable type may also regulate the plot-level variances. Nevertheless, with such variability for SOC and TN in unfertilized plots, three samples per plot will produce a relative error of the mean of about ± 20% in both bioenergy croplands, a sobering result given the level of interest in precise estimates of soil C and N sequestrations.

### N fertilization restructured the spatial patterns of SOC and TN

Plowing, fertilization and irrigation are common management practices in cultivated row crops, which can homogenize a wide range of soil physiochemical characteristics^21,55^. The results of the current study supported that lack of other common management practices, N fertilizer input alone however reestablished spatial pattern and structure of SOC and TN, i.e., more significant surface trends in various directions and more pronounced spatial autocorrelations in the fertilized plots. This seemingly contradiction suggested that in the traditional croplands, the mechanical management practices, e.g., tillage and irrigation, acted as dominantly physical agents, which potentially overwhelmed the impacts of other chemical agents such as fertilizers. Besides N fertilizer input, minimal mechanical disturbance and no other chemical agents were amended in the research plots of the current study. Despite the indigenous plot level variations, the N fertilization input as a sole chemical agent, produced an overwhelming effect over the indigenous irregularity of soil nitrogen and likely other nutrient availability^20^ and reconstructed new spatial structure and pattern.

Another possible explanation is that N fertilizer enhanced the number of hot spots by stimulating microbial activities and turnover^56^. Consequently, soil fungal hyphae might migrate from root zone to the emerging hot spots^57^. The emerging hot spots and associated root and microbial activities together may have restructured spatial variations beyond the root zone, leading to overall greater spatial heterogeneity in microbial biomass^22^, and consequently on SOC and TN. Indeed, our analyses demonstrated significantly positive correlations between SOC, TN and microbial biomass (Table S2). Our data corroborated the restructuring of spatial patterns in both soil bulk C and nutrients storage at the rhizosphere and beyond^58^.

### N fertilization rate little impacted plot-level variation and spatial heterogeneity

Though significantly different from the control treatment, the low and high fertilization treatments appeared little different in either the plot-level variation or spatial heterogeneity in SOC and TN. We speculated that the experimental duration may have contributed to this phenomenon. The current study relied upon a three-year long experiment, but a longer N fertilization history (e.g., > 10 years) may allow us to detect the appreciable changes in spatial heterogeneity under N fertilizations. The reason lied in the fact that the changes in soil C storage were more likely detectable over decades in long-term soil experiments^59,60^. On the other hand, our former synthesis study demonstrated that multiple soil extracellular enzyme activities were enhanced with a longer fertilization history when compared between the experiments with <1 year and 1~10 years, and those with >10 years^61^. As soil exoenzymes are the proximate agent of soil decomposition, the more pronounced changes in soil exoenzyme activities over more than 10 years may inform the potentially appreciable change in SOC and TN over longer time. Nevertheless, the magnitude of fertilizer input may also play a role because the fertilization rate in the current study (i.e., 84–168 kg N ha^−1^) is below the high fertilization rate (e.g., >200 kg N ha^−1^) applied in other croplands worldwide^62^. Long-term soil studies will need to specifically include the spatial variability in their research theme in the future.

### Mixed effects of N fertilization on the spatial patterns of C: N, δ^13^C, and δ^15^N

Relative to SOC and TN, the C: N, δ^13^C, and δ^15^N displayed relatively lower plot-level variations under all fertilization treatments in both croplands. This suggested that these specific variables, i.e., derived from other measured variables or proxy of integrated ecosystem processes, likely masked the inherently high variation of each individual process. Nevertheless, the fertilization effects on their spatial patterns depend on the type of geostatistical analysis, crop type and variable. Almost no surface trend was identified for C: N and δ^13^C in any plot in SG, which contrasted substantially with their counterparts in GG. Both surface trends and spatial autocorrelations for δ^15^N were much abundant in both croplands. However, no fertilization effects were significant in these analyses. In particular, the higher N fertilizer input resulted in no any significant surface trends of either δ^13^C or δ^15^N in all plots in GG. Meanwhile, great plot to plot variations were identified between the distribution maps of two unfertilized plots for C: N and δ^13^C, and these variations were reduced in fertilized plots, a fertilization effect consistent with those found in SOC and TN. Despite low plot-level variation, the high plot-to-plot variations between the two unfertilized plots for C: N and δ^13^C may result from a suite of interactions along the air-plant-soil-microbe continuum involved in photosynthate, root exudate, microbe-root associations, and other edaphic features. These multiple drivers themselves entailed high spatial heterogeneities in soils over various spatial scales^21,22,63–66^. Therefore, the complexity of biogeochemistry in soils likely has masked the effect of N fertilization on C: N, ^13^C, and ^15^N in at least some of their spatial patterns. Despite this uncertainty, the distribution maps produced in this study enabled us to detect fertilization effects that were similar across all these variables.

## Conclusions

Our study demonstrated that N fertilizer input generally diminished the plot-level variances of all five variables in SG cropland but re-established the spatial structures of SOC and TN in both croplands. Although N fertilization apparently reduced the plot to plot difference in distribution maps of C: N, δ^13^C, and δ^15^N, little fertilization effect was identified on their surface trends and spatial autocorrelations due to either weak or strong patterns simultaneously identified in unfertilized and fertilized plots. Effects of  low and high rates of fertilization were found identical in this study. The descending within-plot variations were also identified among variables (SOC > TN > δ^15^N > C: N > δ^13^C). This study demonstrated that distinct from the homogenization of spatial structures caused by multiple practices in traditional croplands (e.g., plowing, irrigation and fertilization), N fertilizations alone generally reduced the plot-level variance and simultaneously re-established spatial structures of SOC and TN in bioenergy croplands, which little varied with fertilization rate but was more responsive in switchgrass cropland.

## Supplementary information

Supplemental Tables&Figures.
