# Supplementary material for: Nitrogen Fertilization Restructured Spatial Patterns of Soil Organic Carbon and Total Nitrogen in Switchgrass and Gamagrass Croplands in Tennessee USA
Source: Sci Rep. 2020 Jan 27;10:1211. doi: 10.1038/s41598-020-58217-x (PMC6985139; doi:10.1038/s41598-020-58217-x)
Supplement: Supplementary file 1 — Supplemental Tables&Figures. [file 41598_2020_58217_MOESM1_ESM.docx]

**Supplemental Materials**

**Nitrogen Fertilization Restructured Spatial Patterns of Soil Organic Carbon and Total Nitrogen in Switchgrass and Gamagrass Croplands** **in Tennessee USA**

Jianwei Li^1*^, Siyang Jian^1^, Chad S. Lane^2^, Chunlan Guo^3^, YueHan Lu^4^, Qi Deng^5^, Melanie A. Mayes^6^, Kudjo E Dzantor^1^, Dafeng Hui^7^

Table S1. The summary statistics of (a) SOC, TN, C: N, and (b) δ^13^C and δ^15^N under three N fertilization (i.e. NN, LN and HN) in (a) SG and (b) GG cropland soils in a three-year long fertilization experimental site at the Tennessee State University (TSU) Agricultural Research Center in Nashville, TN, USA. The abbreviations are referred to Table 1. (N=48)

(a)

(b)

Table S2. The Pearson Moment correlation coefficients among SOC, TN, C: N, microbial biomass carbon (MBC), microbial biomass N (MBN) and MBC: MBN (C: N_mb_) in (a) SG and GG, (b) SG and (c) GG. Bold numbers denote significant correlation coefficients at *P* < 0.05.

**Figure S1.** Frequency histograms of SOC and TN, C:N, δ^13^C, and δ^15^N under each of three fertilization treatments (i.e. NN, LN and HN) in SG (panels in left column) and GG (panels in right column) croplands in a three-year long fertilization experimental site at the Tennessee State University (TSU) Agricultural Research Center in Nashville, TN, USA. Data of two replicated plots were pooled together in each panel. The number on the x-axis (i.e. 1, 1.5 in top left panel) represents a range of (0.5, 1) and (1, 1.5), respectively.

**Figure S2.** Moran^’^s *I* correlograms for (a) SOC, (b) TN, (c) C:N, (d) δ^13^C, and (e) δ^15^N in two plots (P1, P2) under three N fertilization treatments (i.e. NN, LN and HN) in SG and GG cropland soils in a three-year long fertilization experimental site at the Tennessee State University (TSU) Agricultural Research Center in Nashville, TN, USA. Filled circles denote Moran^’^s *I* values that exhibited significant positive or negative autocorrelation. Obs: observations; LCL: low confident limit; and UCL: Upper confidence limit.
